# Supplementary material for: Diet and Risk of Non-Alcoholic Fatty Liver Disease, Cirrhosis, and Liver Cancer: A Large Prospective Cohort Study in UK Biobank
Source: Nutrients. 2022 Dec 15;14(24):5335. doi: 10.3390/nu14245335 (PMC9788291; doi:10.3390/nu14245335)
Supplement: Supplementary file 1 [file nutrients-14-05335-s001.zip › nutrients-2020995-supplementary.pdf]

Contents

Supplementary Figures:

Supplementary Figure S1. Flowchart for selection of study participants.

Supplementary Figure S2. Correlations between different dietary components.

Supplementary Tables:

Supplementary Table S1. ICD-10 and ICD-9 codes used to define baseline liver disease in UK Biobank.

Supplementary Table S2. Coding and categories of intake for each food group.

Supplementary Table S3. ICD-10 codes used to define liver-related outcomes in UK Biobank.

Supplementary Table S4. Associations between dietary patterns (tertiles) and risk of different types of cirrhosis.

Supplementary Table S5. Associations between food groups and the risk of different liver diseases in UK Biobank.

Supplementary Table S6. Associations between sources of fiber and the risk of different liver diseases in UK Biobank.

Supplementary Table S7. Associations of dietary patterns with incident NAFLD by stratification in the UK Biobank.

Supplementary Table S8. Associations of dietary patterns with incident cirrhosis by stratification in the UK Biobank.

Supplementary Table S9. Associations of dietary patterns with incident liver cancer by stratification in the UK Biobank.

Supplementary Table S10. Associations between dietary patterns (tertiles) and risk of different liver diseases in participants who did not report changing their diet.

Supplementary Table S11. Associations between dietary patterns (tertiles) and risk of different liver diseases in participants whose follow-up time >3 years.

Supplementary Table S12. Associations between dietary patterns (tertiles) and risk of different liver diseases in participants without excessive alcohol consumption.

Supplementary Table S13. Associations between sources of fiber and the risk of different liver diseases in participants who did not report changing their diet.

Supplementary Table S14. Associations between sources of fiber and the risk of different liver diseases in participants whose follow-up time >3 years.

Supplementary Table S15. Associations between sources of fiber and the risk of different liver diseases in participants in participants without excessive alcohol consumption.

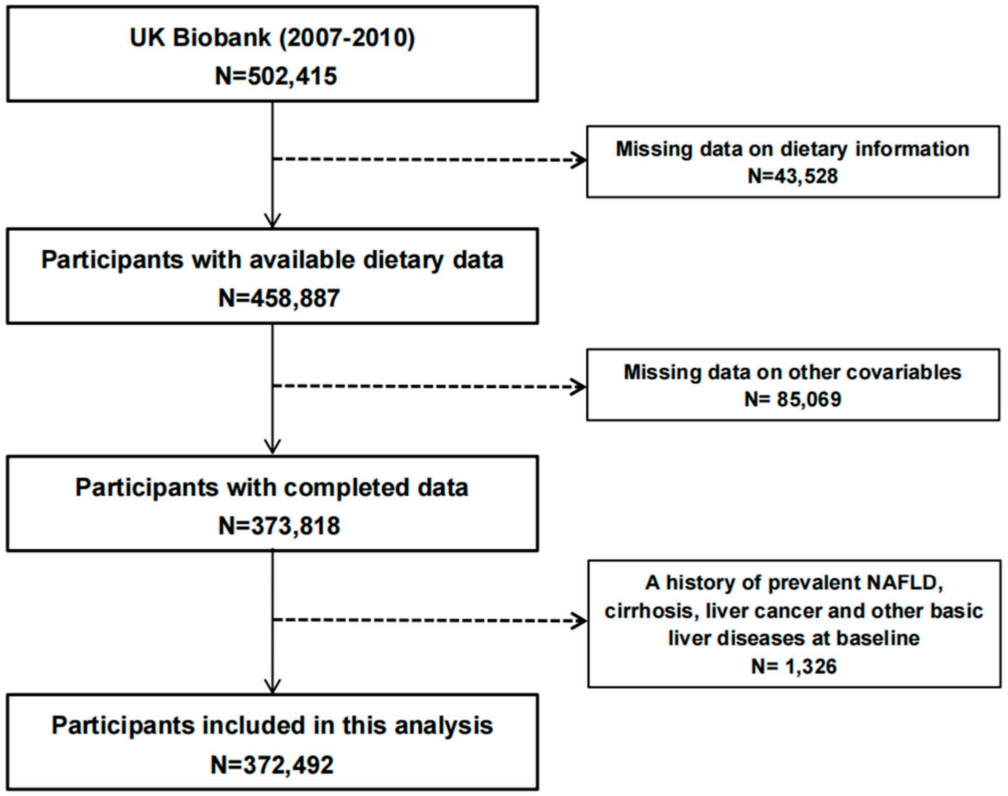

Supplementary Figure S1. Flowchart for selection of study participants.

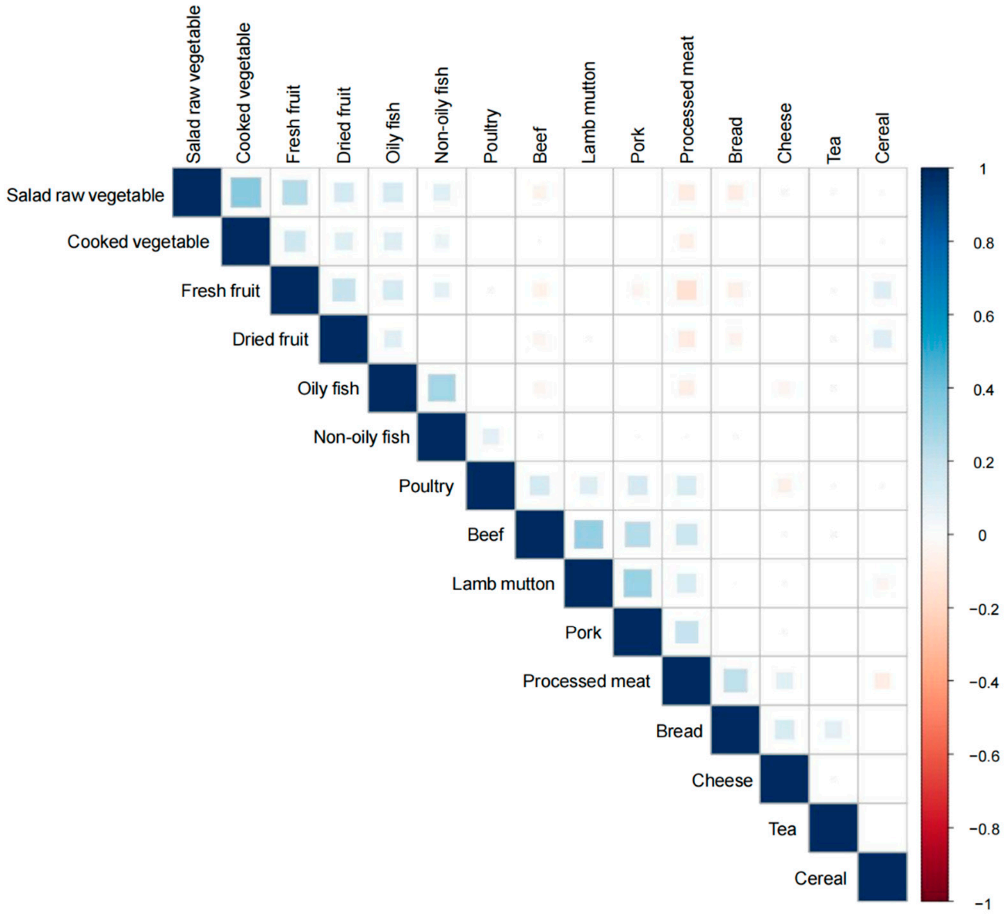

Supplemental Figure S2. Correlations between different dietary components.

Supplemental Table S1. ICD-10 and ICD-9 codes used to define baseline liver disease in UK Biobank.

Disease	ICD-10	ICD-9
Liver cancer	C22	155.0-155.3
Non-alcoholic fatty liver disease	K76.0	571.8
Hepatic fibrosis and cirrhosis	K74	571.5, 571.6
Alcoholic liver disease	K70	571.0, 571.1, 571.2, 571.3
Portal hypertension	K76.6	572.3
Esophageal varices	I85.0, I85.9	456.0, 456.1
Hepatorenal syndrome	K76.7	572.4
Liver failure	K72.1, K72.9	573.8
Viral hepatitis	B16-19	070
Liver transplant status	Z94.4	V42.7

Definition of abbreviations: ICD, International Classification of Diseases.

Supplemental Table S2. Coding and categories of intake for each food group.

Food groups	Components	Coding	Categories
Red meat	Beef	‘Never’ = 0, ‘Less than once a week’ = 0.5, ‘Once a week’ = 1, ‘2-4 times a week’ = 3, ‘5-6 times a week’ = 5.5, ‘Once or more daily’ = 7	<1 time/week, 1.0-1.9 times/week, 2.0-2.9 times/week, and ≥3.0 times/week
	Pork		
	Lamb/mutton		
Processed meat	Processed meat		never, <1.0 time/week, 1.0 time/week, and ≥2.0 times/week
Poultry	Poultry		never, <1.0 time/week, 1.0 time/week, and ≥2.0 times/week
Total fish	Oily fish		<1.0 time/ week, 1.0-1.9 times/week, 2.0-2.9 times/week, and ≥3.0 times/week
	Non-oily fish		
Cheese	Cheese		<1.0 time/week, 1.0 time/week, 2.0-4.9 times/ week, and ≥5.0 times/week
Fruit	Fresh fruit	‘Less than once a week’ = 0.5; Amount per serving: fresh fruit – 1piece; dried fruit – 2 pieces	<2.0 servings/day, 2.0-2.9 servings/day, 3.0-3.9 servings/day, and ≥4.0 servings/day
	Dried fruit		
Vegetables	Cooked vegetables	‘Less than once a week’ = 0.5; Amount per serving: cooked/raw vegetables – 2 heaped tablespoons	<2.0 servings/day, 2.0-2.9 servings/day, 3.0-3.9 servings/day, and ≥4.0 servings/day
	Salad/raw vegetables		
Cereal	Cereal	‘Less than once a week’ = 0.5; Amount per serving: Bran/oat/muesli cereal– 1 bowl/day	<2.0 servings/day, 2.0-4.9 servings/day, 5.0-6.9 servings/day, and ≥7.0 servings/day
Bread	Bread	‘Less than once a week’ = 0.5; Amount per serving: Whole-meal/wholegrain bread – 1 slice/day	<5.0 servings/day, 5.0-9.9 servings/day, 10.0-13.9 servings/day, and ≥14.0 servings/day

Tea	Tea	‘Less than once a week’ = 0.5	<2.0 cups/day, 2.0-3.9 cups/day, 4.0-5.9 cups/day, and ≥6.0 cups/day

Supplemental Table S3. ICD-10 used to define liver-related outcomes in UK Biobank.

Outcome	ICD-10	Case (%)	Definition
Liver cancer	C22	669 (100)	Liver cancer
NAFLD	K76.0	3527 (100)	Non-alcoholic fatty liver disease
Cirrhosis	K74.0	737 (44.85)	Hepatic fibrosis
	K74.1		Hepatic sclerosis
	K74.2		Hepatic fibrosis with hepatic sclerosis
	K74.6		Other and unspecific cirrhosis of liver
	K70.2	314 (19.11)	Alcoholic fibrosis and sclerosis of the liver
	K70.3		Alcoholic cirrhosis
	K70.4		Alcoholic hepatic failure
	K76.6	331 (20.15)	Portal hypertension
	I85.0	261 (15.89)	Esophageal varices, bleeding
	I85.9		Esophageal varices, not bleeding

Definition of abbreviations: ICD, International Classification of Diseases; NAFLD, non-alcoholic fatty liver disease.

Supplemental Table S4. Associations between dietary patterns (tertiles) and risk of different types of cirrhosis.

Dietary pattern	Hepatic fibrosis and cirrhosis[‡]			Alcoholic cirrhosis[‡]			Portal hypertension[‡]			Esophageal varices[‡]		
	Total No. (cases)	HR (95%CI)	*P* value	Total No. (cases)	HR (95%CI)	*P* value	Total No. (cases)	HR (95%CI)	*P* value	Total No. (cases)	HR (95%CI)	*P* value
**Western pattern**												
Tertile 1	124164 (199)	1.00 (reference)	reference	124164 (74)	1.00 (reference)	reference	124164 (79)	1.00 (reference)	reference	124164 (78)	1.00 (reference)	reference
Tertile 2	124164 (224)	0.96 (0.79-1.16)	0.665	124164 (87)	0.98 (0.72-1.34)	0.899	124164 (117)	1.31 (0.98-1.74)	0.068	124164 (75)	0.88 (0.64-1.21)	0.429
Tertile 3	124164 (314)	1.12 (0.93-1.34)	0.231	124164 (153)	1.38 (1.04-1.84)	0.026	124164 (135)	1.26 (0.95-1.68)	0.109	124164 (108)	1.13 (0.84-1.53)	0.422
P trend		0.100			0.012			0.143			0.360	
per 1 SD increase		1.11 (1.04-1.19)	0.001		1.15 (1.04-1.26)	0.004		1.15 (1.05-1.27)	0.003		1.06 (0.95-1.20)	0.305
**Prudent pattern**												
Tertile 1	124164 (287)	1.00 (reference)	reference	124164 (155)	1.00 (reference)	reference	124164 (122)	1.00 (reference)	reference	124164 (101)	1.00 (reference)	reference
Tertile 2	124164 (221)	0.88 (0.73-1.05)	0.149	124164 (83)	0.74 (0.57-0.97)	0.032	124164 (118)	1.13 (0.88-1.47)	0.338	124164 (90)	0.96 (0.72-1.28)	0.758
Tertile 3	124164 (229)	0.91 (0.76-1.09)	0.315	124164 (76)	0.76 (0.57-1.01)	0.055	124164 (91)	0.89 (0.67-1.18)	0.427	124164 (70)	0.75 (0.54-1.02)	0.069
P trend		0.295			0.034			0.477			0.074	
per 1 SD increase		0.94 (0.87-1.02)	0.135		0.84 (0.74-0.96)	0.009		0.92 (0.82-1.04)	0.166		0.87 (0.76-1.00)	0.044

Definition of abbreviations: HR, hazard ratio; 95%CI, 95% confidence interval; SD, standard deviation.

[‡]HRs and 95% CIs were adjusted for age, sex, race, education level, Townsend Deprivation Index (quartiles), drinking status, smoking status, exercise, BMI and diabetes.

Supplemental Table S5. Associations between food groups and the risk of different liver diseases in UK Biobank.

Food group	NAFLD‡			Cirrhosis‡			Liver cancer‡		
	Total No. (cases)	HR (95%CI)	*P* value	Total No. (cases)	HR (95%CI)	*P* value	Total No. (cases)	HR (95%CI)	*P* value
**Red meat**									
<1.0 times/week	36648 (269)	1.00 (reference)	reference	36648 (118)	1.00 (reference)	reference	36648 (44)	1.00 (reference)	reference
1.0-1.9 times/week	147394 (1345)	1.10 (0.96-1.26)	0.157	147394 (579)	1.00 (0.82-1.23)	0.972	147394 (240)	1.07 (0.77-1.48)	0.683
2.0-2.9 times/week	106225 (993)	1.09 (0.95-1.25)	0.204	106225 (432)	0.97 (0.79-1.19)	0.754	106225 (190)	1.08 (0.77-1.50)	0.668
≥3.0 times/week	82225 (920)	1.18 (1.03-1.35)	0.021	82225 (514)	1.30 (1.06-1.60)	0.011	82225 (195)	1.29 (0.93-1.81)	0.130
P trend		0.030			1.14E-04			0.045	
**Processed meat**									
<1.0 time/week	147721 (1204)	1.00 (reference)	reference	147721 (511)	1.00 (reference)	reference	147721 (228)	1.00 (reference)	reference
1.0-2.9 time/week	109103 (1029)	1.06 (0.97-1.15)	0.216	109103 (453)	0.99 (0.87-1.13)	0.903	109103 (191)	0.98 (0.81-1.20)	0.863
3.0-4.9 time/week	101196 (1122)	1.14 (1.04-1.24)	0.003	101196 (569)	1.13 (1.00-1.28)	0.058	101196 (220)	1.11 (0.92-1.35)	0.276
≥5.0 times/week	14472 (172)	1.17 (0.99-1.38)	0.058	14472 (110)	1.35 (1.09-1.67)	0.005	14472 (30)	1.02 (0.69-1.51)	0.909
P trend		0.002			0.004			0.355	
**Poultry**									
Never	18852 (116)	1.00 (reference)	reference	18852 (64)	1.00 (reference)	reference	18852 (16)	1.00 (reference)	reference
<1.0 time/week	38724 (329)	1.17 (0.95-1.45)	0.149	38724 (195)	1.07 (0.81-1.42)	0.635	38724 (81)	1.64 (0.96-2.82)	0.071
1.0 time/week	133697 (1187)	1.21 (1.00-1.47)	0.049	133697 (605)	1.03 (0.79-1.33)	0.849	133697 (253)	1.58 (0.95-2.62)	0.080
≥2.0 times/week	181219 (1895)	1.33 (1.10-1.61)	0.003	181219 (779)	1.02 (0.79-1.32)	0.899	181219 (319)	1.66 (1.00-2.75)	0.050
P trend		1.24E-04			0.728			0.186	
**Total fish**									
<1.0 time/week	26732 (306)	1.00 (reference)	reference	26732 (126)	1.00 (reference)	reference	26732 (42)	1.00 (reference)	reference
1.0-1.9 times/week	149169 (1422)	0.92 (0.81-1.04)	0.205	149169 (661)	0.98 (0.81-1.18)	0.811	149169 (267)	1.00 (0.72-1.38)	0.984
2.0-2.9 times/week	90356 (771)	0.87 (0.76-1.00)	0.045	90356 (387)	0.98 (0.80-1.20)	0.833	90356 (152)	0.87 (0.61-1.23)	0.421
≥3.0 times/week	106235 (1028)	0.95 (0.83-1.08)	0.420	106235 (469)	0.97 (0.80-1.18)	0.771	106235 (208)	0.98 (0.70-1.37)	0.915
P trend		0.777			0.828			0.737	
**Cheese**									
<1.0 time/week	71162 (787)	1.00 (reference)	reference	71162 (331)	1.00 (reference)	reference	71162 (142)	1.00 (reference)	reference
1.0 time/week	78407 (827)	1.01 (0.91-1.11)	0.900	78407 (348)	0.97 (0.83-1.12)	0.651	78407 (144)	0.91 (0.72-1.14)	0.409

2.0-4.9 times/week	171326 (1532)	0.94 (0.86-1.02)	0.134	171326 (767)	1.03 (0.91-1.18)	0.619	171326 (297)	0.90 (0.73-1.10)	0.291
≥5.0 times/week	51597 (381)	0.88 (0.77-0.99)	0.037	51597 (197)	0.97 (0.81-1.16)	0.725	51597 (86)	0.95 (0.72-1.24)	0.684
P trend		0.015			0.818			0.506	
**Fruit**									
<2.0 servings/day	120431 (1294)	1.00 (reference)	reference	120431 (673)	1.00 (reference)	reference	120431 (256)	1.00 (reference)	reference
2.0-2.9 servings/day	95555 (862)	0.90 (0.82-0.98)	0.016	95555 (377)	0.80 (0.71-0.91)	7.34E-04	95555 (156)	0.78 (0.64-0.95)	0.015
3.0-3.9 servings/day	73186 (609)	0.82 (0.75-0.91)	9.90E-05	73186 (263)	0.74 (0.64-0.86)	6.91E-05	73186 (106)	0.68 (0.54-0.86)	0.001
≥4.0 servings/day	83320 (762)	0.89 (0.81-0.98)	0.015	83320 (330)	0.80 (0.70-0.92)	1.00E-03	83320 (151)	0.82 (0.67-1.01)	0.065
P trend		0.002			1.60E-04			0.021	
**Vegetables**									
<2.0 servings/day	127445 (1283)	1.00 (reference)	reference	127445 (632)	1.00 (reference)	reference	127445 (217)	1.00 (reference)	reference
2.0-2.9 servings/day	127715 (1145)	0.95 (0.88-1.03)	0.299	127715 (530)	0.94 (0.83-1.05)	0.266	127715 (243)	1.11 (0.92-1.33)	0.275
3.0-3.9 servings/day	66542 (575)	0.88 (0.80-0.97)	0.013	66542 (278)	0.93 (0.81-1.07)	0.328	66542 (114)	0.98 (0.78-1.24)	0.882
≥4.0 servings/day	50790 (524)	1.03 (0.93-1.14)	0.622	50790 (203)	0.88 (0.75-1.03)	0.104	50790 (95)	1.09 (0.85-1.39)	0.485
P trend		0.565			0.093			0.702	
**Cereal**									
<2.0 servings/week	76608 (849)	1.00 (reference)	reference	76608 (464)	1.00 (reference)	reference	76608 (158)	1.00 (reference)	reference
2.0-4.9 servings/week	74978 (872)	0.99 (0.90-1.09)	0.886	74978 (370)	0.82 (0.72-0.95)	0.006	74978 (134)	0.87 (0.69-1.09)	0.227
5.0-6.9 servings/week	78073 (707)	0.87 (0.79-0.97)	0.008	78073 (303)	0.72 (0.62-0.84)	1.33E-05	78073 (126)	0.83 (0.65-1.05)	0.112
≥7.0 servings/week	142833 (1099)	0.81 (0.74-0.89)	9.71E-06	142833 (506)	0.65 (0.57-0.74)	4.64E-11	142833 (251)	0.78 (0.63-0.95)	0.015
P trend		3.93E-07			2.16E-11			0.017	
**Bread**									
<5.0 servings/week	60763 (552)	1.00 (reference)	reference	60763 (207)	1.00 (reference)	reference	60763 (84)	1.00 (reference)	reference
5.0-9.9 servings/week	84593 (797)	0.98 (0.87-1.09)	0.656	84593 (387)	1.11 (0.94-1.32)	0.224	84593 (145)	1.02 (0.78-1.34)	0.872
10.0-13.9 servings/week	75268 (724)	0.96 (0.86-1.08)	0.520	75268 (317)	0.90 (0.75-1.08)	0.253	75268 (120)	0.90 (0.68-1.19)	0.453
≥14.0 servings/week	151868 (1454)	0.93 (0.84-1.03)	0.145	151868 (732)	0.89 (0.76-1.05)	0.157	151868 (320)	1.03 (0.81-1.32)	0.796
P trend		0.112			0.006			0.801	
**Tea**									
<2.0 cups/day	97772 (1043)	1.00 (reference)	reference	97772 (494)	1.00 (reference)	reference	97772 (189)	1.00 (reference)	reference
2.0-3.9 cups/day	110678 (1012)	0.98 (0.90-1.07)	0.634	110678 (503)	0.99 (0.87-1.12)	0.884	110678 (212)	0.97 (0.79-1.18)	0.747
4.0-5.9 cups/day	94888 (863)	0.97 (0.88-1.06)	0.478	94888 (359)	0.81 (0.71-0.93)	0.002	94888 (152)	0.79 (0.64-0.98)	0.032
≥6.0 cups/day	69154 (609)	0.85 (0.77-0.94)	0.002	69154 (287)	0.79 (0.68-0.91)	0.001	69154 (116)	0.80 (0.63-1.01)	0.059

P trend	0.005	7.30E-05	0.014

Definition of abbreviations: NAFLD, non-alcoholic fatty liver disease; HR, hazard ratio; 95%CI, 95% confidence interval.

‡HRs and 95% CIs were adjusted for age, sex, race, education level, Townsend Deprivation Index (quartiles), drinking status, smoking status, exercise, BMI and diabetes.

Supplemental Table S6. Associations between sources of fiber and the risk of different liver diseases in UK Biobank.

Fiber sources*	NAFLD‡			Cirrhosis‡			Liver cancer‡		
	Total No. (cases)	HR (95%CI)	*P* value	Total No. (cases)	HR (95%CI)	*P* value	Total No. (cases)	HR (95%CI)	*P* value
**Dietary fiber**									
Tertile 1	124174 (1392)	1.00 (reference)	reference	124174 (716)	1.00 (reference)	reference	124174 (240)	1.00 (reference)	reference
Tertile 2	124403 (1094)	0.86 (0.79-0.93)	2.63E-04	124403 (502)	0.78 (0.69-0.88)	2.71E-05	124403 (214)	0.87 (0.72-1.05)	0.139
Tertile 3	123915 (1041)	0.83 (0.76-0.90)	6.98E-06	123915 (425)	0.64 (0.57-0.73)	3.18E-12	123915 (215)	0.83 (0.69-1.01)	0.062
P trend		4.88E-06			1.59E-12			0.062	
Per 5 g/day		0.93 (0.91-0.96)	7.03E-07		0.86 (0.83-0.90)	2.73E-11		0.91 (0.85-0.98)	0.007
**Fiber from bread and cereal**									
Tertile 1	124260 (1447)	1.00 (reference)	reference	124260 (695)	1.00 (reference)	reference	124260 (252)	1.00 (reference)	reference
Tertile 2	124070 (1161)	0.87 (0.80-0.94)	3.54E-04	124070 (516)	0.76 (0.68-0.86)	4.14E-06	124070 (223)	0.83 (0.69-1.00)	0.044
Tertile 3	124162 (919)	0.75 (0.69-0.82)	4.40E-11	124162 (432)	0.64 (0.56-0.72)	2.12E-12	124162 (194)	0.70 (0.57-0.85)	2.68E-04
P trend		3.26E-11			9.41E-13			2.58E-04	
Per 5 g/day		0.81 (0.76-0.87)	1.45E-10		0.70 (0.64-0.77)	1.01E-13		0.78 (0.68-0.90)	5.75E-04
**Fiber from fruit**									
Tertile 1	125637 (1329)	1.00 (reference)	reference	125637 (683)	1.00 (reference)	reference	125637 (261)	1.00 (reference)	reference
Tertile 2	140987 (1299)	0.93 (0.86-1.01)	0.073	140987 (576)	0.88 (0.78-0.98)	0.026	140987 (237)	0.82 (0.69-0.98)	0.033
Tertile 3	105868 (899)	0.89 (0.81-0.97)	0.009	105868 (384)	0.84 (0.73-0.96)	0.009	105868 (171)	0.79 (0.64-0.97)	0.025
P trend		0.008			0.006			0.019	
Per 5 g/day		0.94 (0.89-0.99)	0.028		0.93 (0.86-1.00)	0.063		0.90 (0.80-1.02)	0.114
**Fiber from vegetables**									
Tertile 1	127445 (1283)	1.00 (reference)	reference	127445 (632)	1.00 (reference)	reference	127445 (217)	1.00 (reference)	reference
Tertile 2	127168 (1138)	0.98 (0.91-1.07)	0.683	127168 (527)	0.99 (0.88-1.11)	0.867	127168 (241)	1.16 (0.96-1.40)	0.123
Tertile 3	117879 (1106)	0.99 (0.91-1.07)	0.764	117879 (484)	0.97 (0.86-1.10)	0.684	117879 (211)	1.10 (0.90-1.34)	0.351
P trend		0.757			0.686			0.342	
Per 5 g/day		1.01 (0.96-1.06)	0.603		0.95 (0.87-1.02)	0.170		1.04 (0.93-1.16)	0.483

Definition of abbreviations: NAFLD, non-alcoholic fatty liver disease; HR, hazard ratio; 95%CI, 95% confidence interval.

‡HRs and 95% CIs were adjusted for age, sex, race, education level, Townsend Deprivation Index (quartiles), drinking status, smoking status, exercise, BMI and diabetes.

*Fiber from fruit fiber (vegetable, bread and cereal analyses), vegetables (fruit, bread and cereal analyses), bread and cereal (fruit and vegetable analyses) were adjusted in the multivariable model.

Supplemental Table S7. Associations of dietary patterns with incident NAFLD by stratification in the UK Biobank‡

Covariate	Total No. (cases)	Western pattern			Prudent pattern		
		HR (95%CI)	*P* value	*P* het	HR (95%CI)	*P* value	*P* het
**Age (years)**				0.091			0.689
<60	216688 (2016)	1.10 (1.06,1.14)	2.18E-06		0.97 (0.93,1.02)	0.193	
≥60	155804 (1511)	1.04 (0.99,1.09)	0.132		0.98 (0.93,1.04)	0.545	
**Sex**				0.339			0.077
Male	176327 (1751)	1.06 (1.02,1.11)	0.006		0.94 (0.90,0.99)	0.020	
Female	196165 (1776)	1.09 (1.04,1.15)	1.46E-04		1.00 (0.96,1.05)	0.879	
**Ethnic background**				0.703			0.999
White	355879 (3342)	1.08 (1.04,1.12)	4.55E-06		0.97 (0.94,1.01)	0.130	
Non-white	16613 (185)	1.06 (0.96,1.17)	0.244		0.97 (0.87,1.08)	0.608	
**Townsend deprivation index**				0.248			0.999
Below median	186233 (1397)	1.10 (1.05,1.16)	2.10E-04		0.97 (0.92,1.03)	0.350	
Above median	186259 (2130)	1.06 (1.02,1.10)	0.002		0.97 (0.93,1.02)	0.202	
**Education**				0.849			0.491
College or University degree	133579 (874)	1.08 (1.02,1.15)	0.014		0.99 (0.93,1.06)	0.842	
No degree	238913 (2653)	1.07 (1.03,1.11)	1.68E-04		0.97 (0.93,1.00)	0.085	
**Regular exercise**				0.991			0.093
Yes	202347 (1623)	1.07 (1.03,1.12)	0.002		1.00 (0.95,1.05)	0.992	
No	170145 (1904)	1.07 (1.03,1.12)	0.001		0.94 (0.90,0.99)	0.023	
**Smoking status**				0.089			0.715
Never	205007 (1581)	1.04 (0.99,1.09)	0.096		0.97 (0.92,1.02)	0.282	
Former	130392 (1424)	1.12 (1.07,1.18)	4.43E-06		0.97 (0.91,1.02)	0.204	
Current	37093 (522)	1.05 (0.97,1.14)	0.207		1.01 (0.92,1.10)	0.879	
**Drinking status**				0.171			0.608
Never	13726 (205)	1.10 (0.99,1.22)	0.063		1.02 (0.91,1.14)	0.725	
Former	11802 (225)	1.18 (1.06,1.31)	0.002		0.94 (0.83,1.07)	0.341	
Current	346964 (3097)	1.06 (1.03,1.10)	0.001		0.97 (0.93,1.01)	0.112	
**BMI**				0.230			0.608
Normal	126020 (357)	1.14 (1.04,1.25)	0.006		0.96 (0.86,1.07)	0.460	
Overweight	160163 (1286)	1.09 (1.03,1.15)	0.001		0.96 (0.90,1.01)	0.130	
Obesity	86309 (1884)	1.05 (1.01,1.09)	0.025		0.99 (0.95,1.04)	0.696	
**Diabetes**				0.024			0.196
Yes	17869 (588)	1.00 (0.93,1.07)	0.919		1.02 (0.94,1.10)	0.649	
No	354623 (2939)	1.09 (1.06,1.13)	2.62E-07		0.96 (0.93,1.00)	0.052	

Definition of abbreviations: HR, hazard ratio; 95%CI, 95% confidence interval; het, heterogeneity.

‡HRs and 95% CIs were estimated using Cox proportional-hazard models with adjustment for age, sex, race, education level, Townsend Deprivation Index (quartiles), drinking status, smoking status, exercise, BMI and diabetes.

Supplemental Table S8. Associations of dietary patterns with incident cirrhosis by stratification in the UK Biobank[‡].

Covariate	Total No. (cases)	Western pattern			Prudent pattern		
		HR (95%CI)	*P* value	*P* het	HR (95%CI)	*P* value	*P* het
**Age (years)**				0.293			0.417
<60	216688 (763)	1.15 (1.08,1.22)	5.71E-06		0.94 (0.87,1.01)	0.115	
≥60	155804 (880)	1.10 (1.03,1.17)	0.004		0.90 (0.84,0.97)	0.005	
**Sex**				0.994			0.808
Male	176327 (1105)	1.12 (1.07,1.18)	7.91E-06		0.90 (0.85,0.96)	0.002	
Female	196165 (538)	1.12 (1.03,1.22)	0.007		0.92 (0.84,1.01)	0.073	
**Ethnic background**				0.317			0.041
White	355879 (1589)	1.13 (1.08,1.18)	1.07E-07		0.90 (0.85,0.95)	1.03E-04	
Non-white	16613 (54)	1.03 (0.85,1.23)	0.788		1.07 (0.91,1.25)	0.421	
**Townsend deprivation index**				0.759			0.497
Below median	186233 (628)	1.13 (1.05,1.22)	0.001		0.88 (0.81,0.97)	0.009	
Above median	186259 (1015)	1.12 (1.06,1.18)	4.23E-05		0.92 (0.86,0.98)	0.011	
**Education**				0.808			0.111
College or University degree	133579 (411)	1.13 (1.04,1.23)	0.005		0.84 (0.75,0.94)	0.002	
No degree	238913 (1232)	1.12 (1.06,1.18)	1.43E-05		0.93 (0.88,0.99)	0.018	
**Regular exercise**				0.642			0.671
Yes	202347 (751)	1.13 (1.07,1.21)	6.50E-05		0.92 (0.85,0.99)	0.024	
No	170145 (892)	1.11 (1.05,1.18)	0.001		0.90 (0.83,0.97)	0.005	
**Smoking status**				0.088			0.111
Never	205007 (626)	1.07 (1.00,1.16)	0.065		0.86 (0.79,0.94)	0.001	
Former	130392 (702)	1.11 (1.04,1.19)	0.003		0.97 (0.90,1.05)	0.447	
Current	37093 (315)	1.21 (1.12,1.32)	6.20E-06		0.88 (0.78,0.99)	0.034	
**Drinking status**				0.213			0.386
Never	13726 (67)	0.93 (0.74,1.16)	0.506		0.88 (0.69,1.12)	0.299	
Former	11802 (123)	1.16 (1.01,1.34)	0.035		1.01 (0.86,1.18)	0.910	
Current	346964 (1453)	1.13 (1.08,1.19)	2.16E-07		0.90 (0.85,0.95)	2.79E-04	
**BMI**				0.222			0.954
Normal	126020 (317)	1.19 (1.09,1.31)	1.32E-04		0.90 (0.79,1.01)	0.077	
Overweight	160163 (591)	1.12 (1.04,1.21)	0.003		0.92 (0.84,1.00)	0.052	
Obesity	86309 (735)	1.08 (1.01,1.15)	0.019		0.92 (0.85,0.99)	0.028	
**Diabetes**				0.905			0.017
Yes	17869 (346)	1.12 (1.02,1.22)	0.016		1.02 (0.92,1.13)	0.743	
No	354623 (1297)	1.12 (1.07,1.18)	5.23E-06		0.88 (0.83,0.94)	4.35E-05	

Definition of abbreviations: HR, hazard ratio; 95%CI, 95% confidence interval; het, heterogeneity.

[‡]HRs and 95% CIs were estimated using Cox proportional-hazard models with adjustment for age, sex, race, education level, Townsend Deprivation Index (quartiles), drinking status, smoking status, exercise, BMI and diabetes.

Supplemental Table S9. Associations of dietary patterns with incident liver cancer by stratification in the UK Biobank‡.

Covariate	Total No. (cases)	Western pattern			Prudent pattern		
		HR (95%CI)	*P* value	*P* het	HR (95%CI)	*P* value	*P* het
**Age (years)**				0.965			0.911
<60	216688 (205)	1.10 (0.97,1.24)	0.156		0.97 (0.84,1.12)	0.698	
≥60	155804 (464)	1.09 (1.00,1.19)	0.051		0.96 (0.87,1.06)	0.430	
**Sex**				0.786			0.056
Male	176327 (412)	1.10 (1.01,1.20)	0.035		0.88 (0.79,0.98)	0.024	
Female	196165 (257)	1.08 (0.95,1.22)	0.265		1.04 (0.92,1.18)	0.558	
**Ethnic background**				0.507			0.695
White	355879 (650)	1.10 (1.02,1.18)	0.017		0.94 (0.86,1.02)	0.156	
Non-white	16613 (19)	0.98 (0.70,1.36)	0.884		1.01 (0.73,1.40)	0.969	
**Townsend deprivation index**				0.545			0.190
Below median	186233 (316)	1.06 (0.95,1.19)	0.311		0.88 (0.77,1.00)	0.059	
Above median	186259 (353)	1.11 (1.01,1.22)	0.029		0.99 (0.89,1.10)	0.797	
**Education**				0.576			0.255
College or University degree	133579 (182)	1.05 (0.92,1.21)	0.460		0.87 (0.73,1.03)	0.100	
No degree	238913 (487)	1.10 (1.02,1.20)	0.021		0.97 (0.88,1.07)	0.539	
**Regular exercise**				0.369			0.726
Yes	202347 (336)	1.05 (0.95,1.17)	0.324		0.96 (0.86,1.07)	0.436	
No	170145 (333)	1.13 (1.02,1.25)	0.021		0.93 (0.82,1.05)	0.245	
**Smoking status**				0.101			0.029
Never	205007 (278)	1.00 (0.88,1.13)	0.988		1.02 (0.90,1.15)	0.757	
Former	130392 (295)	1.10 (0.99,1.23)	0.071		0.96 (0.85,1.08)	0.460	
Current	37093 (96)	1.24 (1.06,1.45)	0.006		0.71 (0.56,0.90)	0.005	
**Drinking status**				0.927			0.031
Never	13726 (29)	1.02 (0.73,1.44)	0.894		1.24 (1.01,1.52)	0.040	
Former	11802 (23)	1.11 (0.79,1.58)	0.539		0.87 (0.59,1.30)	0.509	
Current	346964 (617)	1.09 (1.01,1.18)	0.022		0.92 (0.85,1.01)	0.077	
**BMI**				0.848			0.598
Normal	126020 (162)	1.10 (0.94,1.28)	0.217		0.90 (0.76,1.07)	0.229	
Overweight	160163 (264)	1.06 (0.94,1.20)	0.328		0.99 (0.87,1.13)	0.898	
Obesity	86309 (243)	1.11 (0.99,1.25)	0.064		0.92 (0.80,1.05)	0.227	
**Diabetes**				0.521			0.562
Yes	17869 (121)	1.04 (0.88,1.22)	0.641		0.99 (0.83,1.18)	0.907	
No	354623 (548)	1.10 (1.02,1.20)	0.017		0.93 (0.85,1.02)	0.137	

Definition of abbreviations: HR, hazard ratio; 95%CI, 95% confidence interval; het, heterogeneity.

‡HRs and 95% CIs were estimated using Cox proportional-hazard models with adjustment for age, sex, race, education level, Townsend Deprivation Index (quartiles), drinking status, smoking status, exercise, BMI and diabetes.

Supplemental Table S10. Associations between dietary patterns (tertiles) and risk of different liver diseases in participants who did not report changing their diet (N=229,374).

Dietary pattern	NAFLD‡			Cirrhosis‡			Liver cancer‡		
	Total No. (cases)	HR (95%CI)	*P* value	Total No. (cases)	HR (95%CI)	*P* value	Total No. (cases)	HR (95%CI)	*P* value
**Western pattern**									
Tertile 1	76094 (432)	1.00 (reference)	reference	76094 (212)	1.00 (reference)	reference	76094 (97)	1.00 (reference)	reference
Tertile 2	76369 (525)	1.09 (0.95-1.23)	0.212	76369 (259)	1.06 (0.89-1.28)	0.510	76369 (111)	1.01 (0.77-1.33)	0.929
Tertile 3	76911 (678)	1.23 (1.08-1.39)	0.001	76911 (373)	1.26 (1.06-1.50)	0.009	76911 (150)	1.22 (0.93-1.58)	0.145
P trend		9.20E-04			0.006			0.123	
per 1 SD increase		1.09 (1.04-1.14)	3.16E-04		1.12 (1.06-1.19)	2.16E-04		1.09 (0.99-1.21)	0.092
**Prudent pattern**									
Tertile 1	85403 (731)	1.00 (reference)	reference	85403 (391)	1.00 (reference)	reference	85403 (157)	1.00 (reference)	reference
Tertile 2	76717 (479)	0.84 (0.75-0.95)	0.004	76717 (256)	0.90 (0.76-1.05)	0.186	76717 (96)	0.69 (0.53-0.89)	0.005
Tertile 3	67254 (425)	0.89 (0.78-1.01)	0.062	67254 (197)	0.83 (0.69-0.99)	0.041	67254 (105)	0.84 (0.65-1.09)	0.198
P trend		0.033			0.036			0.139	
per 1 SD increase		0.94 (0.90-1.00)	0.033		0.92 (0.86-0.99)	0.028		0.90 (0.80-1.01)	0.082

Definition of abbreviations: NAFLD, non-alcoholic fatty liver disease; HR, hazard ratio; 95%CI, 95% confidence interval; SD, standard deviation.

‡HRs and 95% CIs were adjusted for age, sex, race, education level, Townsend Deprivation Index (quartiles), drinking status, smoking status, exercise, BMI and diabetes.

Supplemental Table S11. Associations between dietary patterns (tertiles) and risk of different liver diseases in participants whose follow-up time >3 years.

Dietary pattern	NAFLD‡			Cirrhosis‡			Liver cancer‡		
	Total No. 369,288 (cases)	HR (95%CI)	*P* value	Total No. 369,456 (cases)	HR (95%CI)	*P* value	Total No. 369,586 (cases)	HR (95%CI)	*P* value
**Western pattern**									
Tertile 1	123211 (876)	1.00 (reference)	reference	123262 (375)	1.00 (reference)	reference	123298 (154)	1.00 (reference)	reference
Tertile 2	123181 (1020)	1.07 (0.97-1.17)	0.168	123251 (450)	1.05 (0.91-1.20)	0.496	123276 (177)	1.00 (0.81-1.25)	0.990
Tertile 3	122896 (1280)	1.20 (1.10-1.32)	3.52E-05	122943 (598)	1.17 (1.02-1.33)	0.021	123012 (237)	1.17 (0.95-1.44)	0.137
P trend		2.38E-05			0.017			0.112	
per 1 SD increase		1.08 (1.05-1.12)	1.99E-06		1.11 (1.06-1.16)	2.75E-05		1.08 (1.00-1.17)	0.046
**Prudent pattern**									
Tertile 1	122896 (1181)	1.00 (reference)	reference	122956 (580)	1.00 (reference)	reference	123006 (214)	1.00 (reference)	reference
Tertile 2	123162 (976)	0.89 (0.82-0.97)	0.010	123206 (443)	0.90 (0.80-1.02)	0.108	123258 (164)	0.78 (0.63-0.96)	0.018
Tertile 3	123230 (1019)	0.93 (0.85-1.01)	0.094	123294 (400)	0.84 (0.73-0.95)	0.008	123322 (190)	0.88 (0.72-1.08)	0.219
P trend		0.087			0.007			0.215	
per 1 SD increase		0.97 (0.94-1.01)	0.113		0.90 (0.85-0.95)	2.42E-04		0.95 (0.87-1.04)	0.231

Definition of abbreviations: NAFLD, non-alcoholic fatty liver disease; HR, hazard ratio; 95%CI, 95% confidence interval; SD, standard deviation.

‡HRs and 95% CIs were adjusted for age, sex, race, education level, Townsend Deprivation Index (quartiles), drinking status, smoking status, exercise, BMI and diabetes.

Supplemental Table S12. Associations between dietary patterns (tertiles) and risk of different liver diseases in participants without excessive alcohol consumption (N=296,289).

Dietary pattern	NAFLD‡			Cirrhosis‡			Liver cancer‡		
	Total No. (cases)	HR (95%CI)	*P* value	Total No. (cases)	HR (95%CI)	*P* value	Total No. (cases)	HR (95%CI)	*P* value
**Western pattern**									
Tertile 1	105655 (831)	1.00 (reference)	reference	105655 (314)	1.00 (reference)	reference	105655 (148)	1.00 (reference)	reference
Tertile 2	99324 (907)	1.06 (0.97-1.17)	0.216	99324 (334)	1.02 (0.87-1.19)	0.820	99324 (166)	1.08 (0.86-1.34)	0.526
Tertile 3	91310 (1018)	1.16 (1.06-1.28)	0.002	91310 (395)	1.12 (0.97-1.31)	0.129	91310 (183)	1.17 (0.94-1.46)	0.160
P trend		0.002			0.121			0.158	
per 1 SD increase		1.07 (1.03-1.11)	1.36E-04		1.08 (1.02-1.15)	0.007		1.02 (0.93-1.12)	0.638
**Prudent pattern**									
Tertile 1	95199 (970)	1.00 (reference)	reference	95199 (374)	1.00 (reference)	reference	95199 (164)	1.00 (reference)	reference
Tertile 2	99727 (858)	0.91 (0.83-0.99)	0.038	99727 (338)	0.96 (0.82-1.11)	0.566	99727 (157)	0.89 (0.71-1.11)	0.286
Tertile 3	101363 (928)	0.95 (0.87-1.04)	0.286	101363 (331)	0.91 (0.78-1.06)	0.243	101363 (176)	0.94 (0.75-1.17)	0.563
P trend		0.290			0.243			0.587	
per 1 SD increase		0.98 (0.94-1.01)	0.202		0.95 (0.89-1.01)	0.124		0.98 (0.89-1.07)	0.634

Definition of abbreviations: NAFLD, non-alcoholic fatty liver disease; HR, hazard ratio; 95%CI, 95% confidence interval; SD, standard deviation.

‡HRs and 95% CIs were adjusted for age, sex, race, education level, Townsend Deprivation Index (quartiles), drinking status, smoking status, exercise, BMI and diabetes.

Supplemental Table S13. Associations between sources of fiber and the risk of different liver diseases in participants who did not report changing their diet (N=229,374).

Fiber sources*	NAFLD‡			Cirrhosis‡			Liver cancer‡		
	Total No. (cases)	HR (95%CI)	*P* value	Total No. (cases)	HR (95%CI)	*P* value	Total No. (cases)	HR (95%CI)	*P* value
**Dietary fiber**									
Tertile 1	80675 (727)	1.00 (reference)	reference	80675 (406)	1.00 (reference)	reference	80675 (136)	1.00 (reference)	reference
Tertile 2	76073 (497)	0.84 (0.75-0.95)	0.004	76073 (256)	0.79 (0.68-0.93)	0.004	76073 (113)	0.89 (0.69-1.15)	0.385
Tertile 3	72626 (411)	0.77 (0.68-0.88)	6.30E-05	72626 (182)	0.60 (0.50-0.72)	3.61E-08	72626 (109)	0.89 (0.68-1.15)	0.363
P trend		3.71E-05			2.33E-08			0.356	
Per 5 g/day		0.90 (0.87-0.95)	7.54E-06		0.84 (0.79-0.90)	6.84E-08		0.88 (0.80-0.97)	0.008
**Fiber from bread and cereal**									
Tertile 1	73967 (697)	1.00 (reference)	reference	73967 (373)	1.00 (reference)	reference	73967 (139)	1.00 (reference)	reference
Tertile 2	75830 (537)	0.85 (0.76-0.95)	0.005	75830 (265)	0.77 (0.65-0.90)	0.001	75830 (112)	0.78 (0.61-1.01)	0.056
Tertile 3	79577 (401)	0.68 (0.60-0.77)	4.45E-09	79577 (206)	0.60 (0.50-0.72)	1.80E-08	79577 (107)	0.71 (0.55-0.93)	0.011
P trend		4.09E-09			1.15E-08			0.011	
Per 5 g/day		0.77 (0.70-0.84)	4.15E-08		0.66 (0.57-0.75)	9.92E-10		0.79 (0.65-0.96)	0.019
**Fiber from fruit**									
Tertile 1	83789 (717)	1.00 (reference)	reference	83789 (404)	1.00 (reference)	reference	83789 (160)	1.00 (reference)	reference
Tertile 2	85851 (565)	0.89 (0.79-0.99)	0.038	85851 (273)	0.82 (0.70-0.96)	0.014	85851 (118)	0.76 (0.60-0.98)	0.031
Tertile 3	59734 (353)	0.87 (0.76-1.00)	0.046	59734 (167)	0.81 (0.67-0.98)	0.032	59734 (80)	0.77 (0.57-1.02)	0.069
P trend		0.028			0.014			0.043	
Per 5 g/day		0.94 (0.87-1.02)	0.149		0.91 (0.81-1.02)	0.097		0.91 (0.76-1.09)	0.312
**Fiber from vegetables**									
Tertile 1	83334 (669)	1.00 (reference)	reference	83334 (346)	1.00 (reference)	reference	83334 (131)	1.00 (reference)	reference
Tertile 2	78775 (494)	0.91 (0.81-1.03)	0.129	78775 (266)	1.01 (0.86-1.19)	0.900	78775 (128)	1.09 (0.85-1.39)	0.516
Tertile 3	67265 (472)	1.01 (0.90-1.15)	0.828	67265 (232)	1.04 (0.87-1.24)	0.673	67265 (99)	0.98 (0.75-1.29)	0.901
P trend		0.952			0.680			0.946	
Per 5 g/day		1.01 (0.93-1.09)	0.827		0.97 (0.86-1.08)	0.581		0.93 (0.78-1.12)	0.470

Definition of abbreviations: NAFLD, non-alcoholic fatty liver disease; HR, hazard ratio; 95%CI, 95% confidence interval.

‡HRs and 95% CIs were adjusted for age, sex, race, education level, Townsend Deprivation Index (quartiles), drinking status, smoking status, exercise, BMI and diabetes.

[*]Fiber from fruit fiber (vegetable, bread and cereal analyses), vegetables (fruit, bread and cereal analyses), bread and cereal (fruit and vegetable analyses) were adjusted in the multivariable model.

Supplemental Table S14. Associations between sources of fiber and the risk of different liver diseases in participants whose follow-up time >3 years.

Fiber sources*	NAFLD‡ (N=369,288)			Cirrhosis‡ (N=369,456)			Liver cancer‡ (N=369,586)		
	Total No. (cases)	HR (95%CI)	*P* value	Total No. (cases)	HR (95%CI)	*P* value	Total No. (cases)	HR (95%CI)	*P* value
**Dietary fiber**									
Tertile 1	122908 (1263)	1.00 (reference)	reference	122957 (621)	1.00 (reference)	reference	123018 (201)	1.00 (reference)	reference
Tertile 2	123390 (976)	0.84 (0.78-0.92)	9.10E-05	123455 (438)	0.79 (0.70-0.89)	1.87E-04	123494 (183)	0.90 (0.73-1.10)	0.285
Tertile 3	122990 (937)	0.82 (0.75-0.89)	6.32E-06	123044 (364)	0.64 (0.56-0.73)	6.42E-11	123074 (184)	0.86 (0.70-1.06)	0.157
P trend		4.10E-06			3.82E-11			0.158	
Per 5 g/day		0.93 (0.90-0.95)	5.30E-07		0.86 (0.82-0.90)	3.51E-10		0.92 (0.85-0.99)	0.019
**Fiber from bread and cereal**									
Tertile 1	123056 (1317)	1.00 (reference)	reference	123103 (598)	1.00 (reference)	reference	123161 (220)	1.00 (reference)	reference
Tertile 2	123030 (1034)	0.85 (0.78-0.92)	1.08E-04	123096 (445)	0.77 (0.68-0.87)	4.57E-05	123141 (181)	0.77 (0.63-0.94)	0.009
Tertile 3	123202 (825)	0.74 (0.68-0.81)	7.35E-11	123257 (380)	0.67 (0.58-0.76)	2.51E-09	123284 (167)	0.68 (0.56-0.84)	3.61E-04
P trend		4.35E-11			1.32E-09			3.27E-04	
Per 5 g/day		0.81 (0.75-0.86)	2.37E-10		0.71 (0.64-0.79)	3.06E-11		0.76 (0.65-0.89)	4.81E-04
**Fiber from fruit**									
Tertile 1	124359 (1205)	1.00 (reference)	reference	124417 (600)	1.00 (reference)	reference	124461 (217)	1.00 (reference)	reference
Tertile 2	139835 (1161)	0.92 (0.84-0.99)	0.036	139909 (500)	0.87 (0.77-0.98)	0.022	139961 (205)	0.86 (0.71-1.05)	0.143
Tertile 3	105094 (810)	0.88 (0.80-0.97)	0.007	105130 (323)	0.80 (0.69-0.92)	0.002	105164 (146)	0.83 (0.66-1.03)	0.095
P trend		0.006			0.002			0.085	
Per 5 g/day		0.93 (0.88-0.99)	0.016		0.91 (0.84-0.99)	0.028		0.92 (0.81-1.05)	0.232
**Fiber from vegetables**									
Tertile 1	126203 (1146)	1.00 (reference)	reference	126269 (548)	1.00 (reference)	reference	126319 (180)	1.00 (reference)	reference
Tertile 2	126142 (1032)	1.00 (0.92-1.09)	0.971	126188 (457)	0.99 (0.87-1.12)	0.880	126238 (207)	1.21 (0.98-1.48)	0.071
Tertile 3	116943 (998)	1.00 (0.91-1.09)	0.952	116999 (418)	0.97 (0.85-1.11)	0.687	117029 (181)	1.14 (0.92-1.41)	0.242
P trend		0.952			0.689			0.237	
Per 5 g/day		1.02 (0.96-1.07)	0.564		0.95 (0.88-1.04)	0.279		1.05 (0.93-1.18)	0.424

Definition of abbreviations: NAFLD, non-alcoholic fatty liver disease; HR, hazard ratio; 95%CI, 95% confidence interval.

‡HRs and 95% CIs were adjusted for age, sex, race, education level, Townsend Deprivation Index (quartiles), drinking status, smoking status, exercise, BMI and diabetes.

[*]Fiber from fruit fiber (vegetable, bread and cereal analyses), vegetables (fruit, bread and cereal analyses), bread and cereal (fruit and vegetable analyses) were adjusted in the multivariable model.

Supplemental Table S15. Associations between sources of fiber and the risk of different liver diseases in participants in participants without excessive alcohol consumption (N=296,289).

Fiber sources*	NAFLD‡			Cirrhosis‡			Liver cancer‡		
	Total No. (cases)	HR (95%CI)	*P* value	Total No. (cases)	HR (95%CI)	*P* value	Total No. (cases)	HR (95%CI)	*P* value
**Dietary fiber**									
Tertile 1	93536 (1030)	1.00 (reference)	reference	93536 (385)	1.00 (reference)	reference	93536 (153)	1.00 (reference)	reference
Tertile 2	99709 (862)	0.86 (0.79-0.95)	1.67E-03	99709 (327)	0.88 (0.76-1.02)	0.080	99709 (168)	0.98 (0.79-1.23)	0.868
Tertile 3	103044 (864)	0.84 (0.76-0.92)	1.49E-04	103044 (321)	0.76 (0.65-0.89)	4.32E-04	103044 (176)	0.94 (0.75-1.18)	0.595
P trend		1.36E-04			4.28E-04			0.590	
Per 5 g/day		0.93 (0.90-0.96)	1.51E-05		0.93 (0.88-0.98)	0.004		0.94 (0.87-1.01)	0.104
**Fiber from bread and cereal**									
Tertile 1	95028 (1095)	1.00 (reference)	reference	95028 (384)	1.00 (reference)	reference	95028 (177)	1.00 (reference)	reference
Tertile 2	98944 (913)	0.87 (0.80-0.96)	0.003	98944 (350)	0.87 (0.75-1.00)	0.057	98944 (168)	0.84 (0.68-1.04)	0.109
Tertile 3	102317 (748)	0.77 (0.70-0.84)	6.86E-08	102317 (309)	0.74 (0.63-0.86)	1.04E-04	102317 (152)	0.71 (0.57-0.89)	0.003
P trend		5.81E-08			1.02E-04			0.003	
Per 5 g/day		0.83 (0.77-0.89)	1.88E-07		0.80 (0.72-0.90)	1.24E-04		0.79 (0.68-0.93)	0.006
**Fiber from fruit**									
Tertile 1	92568 (957)	1.00 (reference)	reference	92568 (359)	1.00 (reference)	reference	92568 (170)	1.00 (reference)	reference
Tertile 2	113842 (1026)	0.92 (0.84-1.01)	0.065	113842 (391)	0.96 (0.83-1.11)	0.573	113842 (191)	0.88 (0.71-1.08)	0.221
Tertile 3	89879 (773)	0.90 (0.81-0.99)	0.037	89879 (293)	0.93 (0.79-1.09)	0.375	89879 (136)	0.76 (0.59-0.96)	0.022
P trend		0.034			0.373			0.022	
Per 5 g/day		0.96 (0.90-1.01)	0.128		0.99 (0.91-1.09)	0.883		0.89 (0.77-1.02)	0.099
**Fiber from vegetables**									
Tertile 1	102613 (1015)	1.00 (reference)	reference	102613 (392)	1.00 (reference)	reference	102613 (153)	1.00 (reference)	reference
Tertile 2	101132 (883)	0.97 (0.89-1.07)	0.548	101132 (340)	0.98 (0.84-1.13)	0.765	101132 (173)	1.16 (0.93-1.45)	0.180
Tertile 3	92544 (858)	0.97 (0.88-1.07)	0.531	92544 (311)	0.94 (0.80-1.10)	0.435	92544 (171)	1.27 (1.01-1.59)	0.043
P trend		0.523			0.438			0.043	
Per 5 g/day		0.99 (0.93-1.05)	0.740		0.96 (0.87-1.06)	0.373		1.10 (0.98-1.23)	0.116

Definition of abbreviations: NAFLD, non-alcoholic fatty liver disease; HR, hazard ratio; 95%CI, 95% confidence interval.

‡HRs and 95% CIs were adjusted for age, sex, race, education level, Townsend Deprivation Index (quartiles), drinking status, smoking status, exercise, BMI and diabetes.

[*]Fiber from fruit fiber (vegetable, bread and cereal analyses), vegetables (fruit, bread and cereal analyses), bread and cereal (fruit and vegetable analyses) were adjusted in the multivariable model.
